# Supplementary material for: Fecal biomarkers to characterize intestinal inflammation among children with medically attended diarrhea across six low-resource settings: Findings from the Enterics for Global Health (EFGH) Shigella surveillance study
Source: PLoS Negl Trop Dis. 2026 Sep 15;20(9):e0014320. doi: 10.1371/journal.pntd.0014320 (PMC13592711; doi:10.1371/journal.pntd.0014320)
Supplement: Supplementary file 1 [file pntd.0014320.s001.docx]

**S1 Appendix**

**Fecal Biomarkers to Characterize Intestinal Inflammation among Children with Medically Attended Diarrhea across Six Low-Resource Settings: Findings from the Enterics for Global Health (EFGH) Shigella Surveillance Study**

Bri’Anna Horne^ǂ^, Brian Otieno Onyando^ǂ^, Henry Badji^ǂ^, Wardah Mujahid^ǂ^, Taufiqur Rahman Bhuiyan^ǂ^, Mutsai Bakali^ǂ^, Junaid Iqbal, Maribel Paredes Olortegui, Patricia Pavlinac, Bubacar E. Ceesay, Olivia Schultes, Sharon M. Tennant, Billy Ogwel, Desiree Witte, Hannah Atlas, Naveed Ahmed, John Benjamin Ochieng, Khandra Sears, Sadia Islam, Queen Saidi, Samba Juma Jallow, Zarfishan Hussain, Paul Garcia Bardales, James A. Platts-Mills, Richard Omore, Farhana Khanam, Md. Parvel Mosharraf, Ousman Secka, Vitumbiko Munthali, Margaret N. Kosek, Maureen Ndalama, Jen Cornick^&^, Md. Tahir Yousafzai^&^, Md. Jahangir Hossain^&^, Catherine Sonye^&^, Firdausi Qadri^&^, Elizabeth T. Rogawski McQuade^&^, Stephanie A. Brennhofer^&^

^ǂ^ co-first authors

^&^ co-senior authors

Correspondence to: Elizabeth T. Rogawski McQuade or Stephanie A. Brennhofer

Supplemental Materials

[Fig A. Pairwise correlations among log_10_ biomarker concentrations among children with medically attended diarrhea by the six sites in the EFGH sub-study.	4](#_Toc239258211)

[Fig B. Density plot characterizing the distribution of log_10_ hemoglobin concentrations among *Shigella-*attributed cases by caregiver or clinician reported visible blood in stool at screening, enrollment or discharge visits or noted in the diarrhea diary among children with medically attended diarrhea across the six sites in the EFGH sub-study.	5](#_Toc239258212)

[Fig C. Density plot characterizing the distribution of log_10_ hemoglobin concentrations among *Shigella*-detected but not attributed cases by caregiver or clinician reported visible blood in stool at screening, enrollment or discharge visits or noted in the diarrhea diary among children with medically attended diarrhea across the six sites in the EFGH sub-study.	6](#_Toc239258213)

[Fig D. Density plot characterizing the distribution of log_10_ hemoglobin concentrations among non-*Shigella* cases by caregiver or clinician reported visible blood in stool at screening, enrollment or discharge visits or noted in the diarrhea diary among children with medically attended diarrhea across the six sites in the EFGH sub-study.	7](#_Toc239258214)

[Table A. Receiver Operating Characteristics (ROC) curve performance of reported blood in stool and log_10_ concentrations of hemoglobin by *Shigella* attribution and detection status among children with medically attended diarrhea across the six sites in the EFGH sub-study.	8](#_Toc239258215)

[Table B. Pathogen prevalence based on qPCR detection (Ct <35) and qPCR attribution of the 26 enteric pathogens among children with medically attended diarrhea across the six sites in the EFGH sub-study.	9](#_Toc239258216)

[Table C. Associations of individual biomarkers with pathogen specific diarrhea etiologies for the 10 most prevalent enteric pathogens considered attributable by qPCR among children with medically attended diarrhea across the six sites in the EFGH sub-study (co-attribution permitted).	10](#_Toc239258217)

[Fig E. Boxplots characterizing the distribution of log_10_ biomarker concentrations by the 10 most prevalent enteric pathogens among children with medically attended diarrhea across the six sites in the EFGH sub-study, excluding episodes with more than one attributable pathogen; i.e., co-attribution not permitted.	11](#_Toc239258218)

[Fig F. Boxplots characterizing the distribution of log_10_ biomarker concentrations with *Shigella* attribution alone or with bacterial, *Cryptosporidium*, or viral co-attribution among children with medically attended diarrhea across the six sites in the EFGH sub-study (co-attribution permitted).	12](#_Toc239258219)

[Fig G. Boxplots characterizing the distribution of log_10_ biomarker concentrations by the 10 most prevalent enteric pathogens among children with medically attended watery diarrhea across the six sites in the EFGH sub-study (co-attribution permitted).	13](#_Toc239258220)

[Fig H. Pairwise correlations among the log_10_ biomarker concentrations among children with medically attended diarrhea by the six sites in the EFGH sub-study.	14](#_Toc239258221)

[Table D. Sensitivity analysis of the associations of individual biomarkers with pathogen specific diarrhea etiologies among children with medically attended diarrhea across the six sites in the EFGH sub-study, including only one enrollment per child (multiple enrollments removed).	16](#_Toc239258222)

[Fig I. Boxplots characterizing the distribution of log_10_ biomarker concentrations by *Shigella* culture positivity status among diarrhea cases among children with medically attended diarrhea across the six sites in the EFGH sub-study.	17](#_Toc239258223)

[Table E. Associations of individual biomarkers with risk factors for shigellosis among children with medically attended diarrhea across the six sites in the EFGH sub-study (co-attribution permitted).	18](#_Toc239258224)

[Fig J. Associations of individual log_10_ biomarkers concentrations with risk factors for shigellosis among children with medically attended diarrhea across the six sites in the EFGH sub-study (*Shigella*-attribution only).	19](#_Toc239258225)

# Fig A. Pairwise correlations among log_10_ biomarker concentrations among children with medically attended diarrhea by the six sites in the EFGH sub-study.

# Fig B. Density plot characterizing the distribution of log_10_ hemoglobin concentrations among *Shigella-*attributed cases by caregiver or clinician reported visible blood in stool at screening, enrollment or discharge visits or noted in the diarrhea diary among children with medically attended diarrhea across the six sites in the EFGH sub-study.

Note: *Shigella* attributed: Ct < 29.5 (whole stool) or 29.8 (rectal swab).

# Fig C. Density plot characterizing the distribution of log_10_ hemoglobin concentrations among *Shigella*-detected but not attributed cases by caregiver or clinician reported visible blood in stool at screening, enrollment or discharge visits or noted in the diarrhea diary among children with medically attended diarrhea across the six sites in the EFGH sub-study.

Note: *Shigella* detected but not attributable: Ct ≥ 29.5 (whole stool) or 29.8 (rectal swab) and < 35.

# Fig D. Density plot characterizing the distribution of log_10_ hemoglobin concentrations among non-*Shigella* cases by caregiver or clinician reported visible blood in stool at screening, enrollment or discharge visits or noted in the diarrhea diary among children with medically attended diarrhea across the six sites in the EFGH sub-study.

Note: *Shigella* not detected: Ct = 35.

# Table A. Receiver Operating Characteristics (ROC) curve performance of reported blood in stool and log_10_ concentrations of hemoglobin by *Shigella* attribution and detection status among children with medically attended diarrhea across the six sites in the EFGH sub-study.

|  | Optimal Cutpoint | Youden’s Index | Sensitivity | Specificity | PPV | NPV | AUC |
| --- | --- | --- | --- | --- | --- | --- | --- |
| Any diarrhea episode | 0.43 | 0.33 | 0.32 | 0.91 | 0.51 | 0.82 | 0.70 |
| *Shigella* attributed | 0.43 | 0.33 | 0.47 | 0.82 | 0.70 | 0.63 | 0.71 |
| *Shigella* detected, not attributed | 0.44 | 0.28 | 0.27 | 0.91 | 0.47 | 0.81 | 0.65 |
| *Shigella* not detected | 0.77 | 0.16 | 0.22 | 0.93 | 0.24 | 0.92 | 0.60 |

PPV: positive predictive value; NPV: negative predictive value; AUC: area under the curve

*Shigella* attributed: Ct < 29.5 (whole stool) or 29.8 (rectal swab).

*Shigella* detected but not attributable: Ct ≥ 29.5 (whole stool) or 29.8 (rectal swab) and < 35.

*Shigella* not detected: Ct = 35.

# Table B. Pathogen prevalence based on qPCR detection (Ct <35) and qPCR attribution of the 26 enteric pathogens among children with medically attended diarrhea across the six sites in the EFGH sub-study.

|  | Prevalence based qPCR detection | Prevalence based on qPCR attribution* |
| --- | --- | --- |
| *Enteroaggregative E. coli* (EAEC) | 60.4 | -- |
| *Shigella* | 36.1 | 22.7 |
| *Campylobacter jejuni/coli* | 33.8 | 2.7 |
| *Campylobacter* *infans* | 30.7 | -- |
| heat-stabile *Enterotoxigenic E. coli* (ST-ETEC) | 25.1 | 11.4 |
| Atypical *Enteropathogenic E. coli* (aEPEC) | 24.3 | -- |
| *Giardia* | 24.3 | -- |
| Adenovirus 40/41 | 18.8 | 3.9 |
| Typical *Enteropathogenic E. coli* (tEPEC) | 18 | 1.6 |
| Rotavirus | 16.8 | 14.2 |
| *Cryptosporidium* | 14.7 | 6.7 |
| Norovirus GII | 13.8 | 3.1 |
| heat-labile *Enterotoxigenic E. coli* (LT-ETEC) | 13.2 | -- |
| Sapovirus | 12.6 | 2.2 |
| *Campylobacter upsalensis* | 12.4 | -- |
| *Enterocytozoon bieneusi* | 8.1 | -- |
| *Helicobacter pylori* | 6.4 | -- |
| Astrovirus | 4.7 | 1.3 |
| *Salmonella* | 3.4 | 1.2 |
| Norovirus GI | 3.3 | -- |
| *Aeromonas* | 3.2 | 0.1 |
| *Vibrio cholerae* | 1.7 | 0.7 |
| *Isospora* | 0.7 | 0.5 |
| *Cyclospora* | 0.5 | 0.4 |
| *Entamoeba histolytica* | 0.3 | 0.2 |

*Attribution cutoffs are pathogen and fecal sample type specific. Specific cutoffs can be found at Yousafzai, et al. *The Lancet Global Health*. 2026.

# Table C. Associations of individual biomarkers with pathogen specific diarrhea etiologies for the 10 most prevalent enteric pathogens considered attributable by qPCR among children with medically attended diarrhea across the six sites in the EFGH sub-study (co-attribution permitted).

|  | Hemoglobin log_10_  Mean (SD) | Lipocalin-2 log_10_  Mean (SD) | Myeloperoxidase log_10_  Mean (SD) | Calprotectin log_10_  Mean (SD) |
| --- | --- | --- | --- | --- |
| *Shigella* | -0.33 (2.07) | 1.33 (0.91) | 3.30 (0.63) | 5.21 (0.72) |
| tEPEC | -1.21 (2.27) | 1.13 (1.17) | 3.06 (0.64) | 4.79 (0.86) |
| *C. jejuni/coli* | -0.82 (1.89) | 1.41 (0.63) | 3.06 (0.72) | 4.89 (0.80) |
| ST-ETEC | -1.45 (2.26) | 1.09 (1.14) | 3.20 (0.71) | 5.01 (0.78) |
| *Cryptosporidium* | -1.61 (2.06) | 1.35 (0.77) | 3.09 (0.66) | 4.81 (0.91) |
| Adenovirus 40/41 | -1.67 (2.26) | 1.11 (0.94) | 2.92 (0.82) | 4.69 (1.02) |
| Astrovirus | -1.84 (2.15) | 1.14 (0.92) | 2.96 (0.60) | 4.86 (0.75) |
| Norovirus GII | -1.85 (2.29) | 1.17 (0.85) | 3.05 (0.76) | 4.76 (0.98) |
| Rotavirus | -1.63 (2.12) | 0.97 (0.94) | 2.85 (0.74) | 4.43 (1.13) |
| Sapovirus | -1.78 (2.24) | 0.95 (1.24) | 3.09 (0.70) | 4.98 (0.72) |

tEPEC: typical *Enteropathogenic E. coli*; *C. jejuni/coli*: *Campylobacter jejuni/coli*; ST-ETEC: heat-stabile *Enterotoxigenic E. coli*

Hemoglobin back-transformed units: μg/g; Lipocalin-2 back-transformed units: μg/g; Myeloperoxidase back-transformed units: ng/mL; Calprotectin back-transformed units: ng/mL.

Note: Data also reported in Fig 3. The back-transformed values were calculated from the full precision log₁₀ values; they cannot be exactly reproduced by back-transforming the rounded log₁₀ means shown here.

# Fig E. Boxplots characterizing the distribution of log_10_ biomarker concentrations by the 10 most prevalent enteric pathogens among children with medically attended diarrhea across the six sites in the EFGH sub-study, excluding episodes with more than one attributable pathogen; i.e., co-attribution not permitted.

tEPEC: typical *Enteropathogenic E. coli*; ST-ETEC: heat-stabile *Enterotoxigenic E. coli*

# Fig F. Boxplots characterizing the distribution of log_10_ biomarker concentrations with *Shigella* attribution alone or with bacterial, *Cryptosporidium*, or viral co-attribution among children with medically attended diarrhea across the six sites in the EFGH sub-study (co-attribution permitted).

Co-bacterial pathogens: typical *Enteropathogenic E. coli* (tEPEC), *Campylobacter jejuni*/*coli*, heat-stabile *Enterotoxigenic E. coli* (ST-ETEC)

Co-viral pathogens: Adenovirus 40/41, Astrovirus, Norovirus GII, Rotavirus, Sapovirus.

Co-attributions were permitted, indicating that more than one pathogen could have been the attributable cause of diarrhea. Therefore, a single child’s data could be represented across multiple bars.

# Fig G. Boxplots characterizing the distribution of log_10_ biomarker concentrations by the 10 most prevalent enteric pathogens among children with medically attended watery diarrhea across the six sites in the EFGH sub-study (co-attribution permitted).

tEPEC: typical *Enteropathogenic E. coli*; ST-ETEC: heat-stabile *Enterotoxigenic E. coli*. Co-attributions were permitted, indicating that more than one pathogen could have been the attributable cause of diarrhea. Therefore, a single child’s data could be represented across multiple pathogen bars.

# Fig H. Pairwise correlations among the log_10_ biomarker concentrations among children with medically attended diarrhea by the six sites in the EFGH sub-study.

tEPEC: typical *Enteropathogenic E. coli*; ST-ETEC: heat-stabile *Enterotoxigenic E. coli*

Note: Data also reported in Table 1.

# Table D. Sensitivity analysis of the associations of individual biomarkers with pathogen specific diarrhea etiologies among children with medically attended diarrhea across the six sites in the EFGH sub-study, including only one enrollment per child (multiple enrollments removed).

|  | Hemoglobin log_10_ Mean Difference  (95% CI) | Lipocalin-2 log_10_ Mean Difference (95% CI) | Myeloperoxidase  log_10_ Mean Difference  (95% CI) | Calprotectin log_10_ Mean Difference  (95% CI) | Hemoglobin  Ratio  (95% CI) | Lipocalin-2  Ratio  (95% CI) | Myeloperoxidase Ratio  (95% CI) | Calprotectin Ratio  (95% CI) |
| --- | --- | --- | --- | --- | --- | --- | --- | --- |
| *Shigella* | 0.36 (0.30, 0.42) | 0.06 (0.04, 0.09) | 0.08 (0.06, 0.10) | 0.10 (0.08, 0.12) | 2.29 (2.02, 2.61) | 1.16 (1.10, 1.23) | 1.20 (1.15, 1.25) | 1.26 (1.20, 1.32) |
| tEPEC | 0.04 (-0.05, 0.12) | -0.02 (-0.05, 0.02) | 0.01 (-0.02, 0.04) | -0.01 (-0.04, 0.03) | 1.09 (0.89, 1.33) | 0.96 (0.88, 1.05) | 1.02 (0.95, 1.09) | 0.99 (0.91, 1.07) |
| *C. jejuni/coli* | 0.10 (0.02, 0.17) | 0.04 (0.01, 0.07) | -0.00 (-0.03, 0.02) | -0.00 (-0.03, 0.03) | 1.25 (1.06, 1.49) | 1.08 (1.01, 1.16) | 0.99 (0.94, 1.04) | 1.00 (0.94, 1.07) |
| ST-ETEC | -0.08 (-0.15, -0.01) | -0.02 (-0.05, 0.01) | 0.03 (0.00, 0.05) | 0.04 (0.01, 0.07) | 0.83 (0.71, 0.97) | 0.95 (0.88, 1.03) | 1.06 (1.01, 1.11) | 1.09 (1.03, 1.16) |
| *Cryptosporidium* | -0.09 (-0.18, 0.01) | 0.06 (0.02, 0.10) | -0.01 (-0.04, 0.02) | -0.03 (-0.07, 0.01) | 0.82 (0.66, 1.03) | 1.14 (1.04, 1.26) | 0.97 (0.91, 1.04) | 0.93 (0.85, 1.02) |
| Adenovirus 40/41 | -0.06 (-0.13, 0.01) | -0.00 (-0.03, 0.03) | -0.03 (-0.05, -0.00) | -0.04 (-0.07, -0.01) | 0.87 (0.74, 1.03) | 1.00 (0.93, 1.07) | 0.94 (0.89, 0.99) | 0.90 (0.84, 0.97) |
| Astrovirus | -0.27 (-0.53, -0.02) | -0.06 (-0.16, 0.04) | -0.09 (-0.17, -0.02) | -0.01 (-0.13, 0.10) | 0.54 (0.30, 0.97) | 0.87 (0.68, 1.09) | 0.80 (0.68, 0.95) | 0.97 (0.74, 1.26) |
| Norovirus GII | -0.27 (-0.42, -0.12) | -0.05 (-0.10, 0.01) | -0.03 (-0.08, 0.02) | -0.08 (-0.15, -0.02) | 0.54 (0.38, 0.76) | 0.90 (0.79, 1.02) | 0.93 (0.82, 1.05) | 0.82 (0.71, 0.95) |
| Rotavirus | -0.15 (-0.25, -0.06) | -0.05 (-0.09, -0.01) | -0.05 (-0.08, -0.02) | -0.10 (-0.15, -0.05) | 0.70 (0.56, 0.87) | 0.89 (0.82, 0.97) | 0.90 (0.83, 0.96) | 0.80 (0.71, 0.89) |
| Sapovirus | -0.16 (-0.30, -0.02) | -0.06 (-0.12, -0.00) | -0.03 (-0.08, 0.01) | -0.00 (-0.05, 0.04) | 0.70 (0.51, 0.96) | 0.87 (0.76, 0.99) | 0.92 (0.84, 1.02) | 0.99 (0.89, 1.11) |

tEPEC: typical enteropathogenic *E. coli*; *C. jejuni/coli*: *Campylobacter jejuni/coli*; ST-ETEC: heat-stable enterotoxigenic *E. coli*

Hemoglobin back-transformed units: μg/g; Lipocalin-2 back-transformed units: μg/g; Myeloperoxidase back-transformed units: ng/mL; Calprotectin back-transformed units: ng/mL.

Note: The back-transformed (antilogged) values were calculated from the full precision log₁₀ values; they cannot be exactly reproduced by back-transforming the rounded log₁₀ mean differences shown here.

# Fig I. Boxplots characterizing the distribution of log_10_ biomarker concentrations by *Shigella* culture positivity status among diarrhea cases among children with medically attended diarrhea across the six sites in the EFGH sub-study.

# Table E. Associations of individual biomarkers with risk factors for shigellosis among children with medically attended diarrhea across the six sites in the EFGH sub-study (co-attribution permitted).

|  | Hemoglobin log_10_ Mean Difference  (95% CI) | Lipocalin-2 log_10_ Mean Difference (95% CI) | Myeloperoxidase  log_10_ Mean Difference  (95% CI) | Calprotectin log_10_ Mean Difference  (95% CI) | Hemoglobin  Ratio  (95% CI) | Lipocalin-2  Ratio  (95% CI) | Myeloperoxidase Ratio  (95% CI) | Calprotectin Ratio  (95% CI) |
| --- | --- | --- | --- | --- | --- | --- | --- | --- |
| Female | -0.14 (-0.39, 0.12) | 0.06 (-0.05, 0.16) | -0.04 (-0.12, 0.04) | -0.06 (-0.15, 0.03) | 0.73 (0.41, 1.31) | 1.14 (0.89, 1.46) | 0.91 (0.75, 1.09) | 0.87 (0.70, 1.07) |
| Age (in months) | -0.03 (-0.05, -0.01) | -0.01 (-0.02, 0.00) | 0.00 (-0.00, 0.01) | 0.01 (-0.00, 0.01) | 0.94 (0.89, 0.98) | 0.98 (0.96, 1.00) | 1.01 (0.99, 1.03) | 1.01 (0.99, 1.03) |
| Fever | 0.35 (0.08, 0.62) | 0.11 (-0.01, 0.24) | 0.06 (-0.03, 0.15) | 0.10 (-0.01, 0.21) | 2.25 (1.21, 4.18) | 1.30 (0.98, 1.74) | 1.14 (0.92, 1.41) | 1.26 (0.98, 1.62) |
| Vomiting | -0.35 (-0.63, -0.07) | -0.04 (-0.16, 0.07) | -0.01 (-0.10, 0.08) | -0.00 (-0.12, 0.11) | 0.45 (0.23, 0.86) | 0.91 (0.70, 1.18) | 0.98 (0.80, 1.21) | 0.99 (0.77, 1.28) |
| Dysentery | 1.11 (0.86, 1.36) | 0.11 (-0.00, 0.23) | 0.05 (-0.04, 0.13) | 0.13 (0.03, 0.24) | 12.82 (7.21, 22.77) | 1.29 (0.99, 1.69) | 1.12 (0.92, 1.36) | 1.36 (1.08, 1.72) |
| MUAC | 0.11 (-0.11, 0.32) | 0.03 (-0.05, 0.11) | -0.02 (-0.09, 0.05) | -0.03 (-0.10, 0.04) | 1.28 (0.78, 2.10) | 1.07 (0.89, 1.29) | 0.96 (0.82, 1.12) | 0.93 (0.79, 1.10) |
| LAZ/HAZ | -0.03 (-0.16, 0.09) | 0.02 (-0.03, 0.08) | 0.04 (-0.00, 0.08) | 0.02 (-0.02, 0.06) | 0.92 (0.69, 1.24) | 1.06 (0.92, 1.21) | 1.09 (1.00, 1.20) | 1.04 (0.95, 1.14) |
| WLZ/WHZ | -0.18 (-0.38, 0.02) | -0.06 (-0.15, 0.02) | 0.00 (-0.07, 0.07) | 0.01 (-0.06, 0.08) | 0.66 (0.42, 1.06) | 0.87 (0.72, 1.05) | 1.00 (0.86, 1.18) | 1.03 (0.88, 1.21) |

MUAC: mid-upper arm circumference; LAZ/HAZ: length-for-age z-score/height-for-age z-score; WLZ/WHZ: weight-for-length z-score/weight-for-height z-score.

Adjusted for country and co-attributable pathogens. Co-attributions were permitted, indicating that more than one pathogen could have been the attributable cause of diarrhea. Therefore, a single child’s data could be represented across multiple pathogen bars.

Hemoglobin back-transformed units: μg/g; Lipocalin-2 back-transformed units: μg/g; Myeloperoxidase back-transformed units: ng/mL; Calprotectin back-transformed units: ng/mL.

Note: Data also reported in Fig 6. The back-transformed (antilogged) values were calculated from the full precision log₁₀ values; they cannot be exactly reproduced by back-transforming the rounded log₁₀ mean differences shown here.

# Fig J. Associations of individual log_10_ biomarkers concentrations with risk factors for shigellosis among children with medically attended diarrhea across the six sites in the EFGH sub-study (*Shigella*-attribution only).

MUAC = mid-upper arm circumference; LAZ/HAZ = length for age z-score/height for age z-score; WHZ = weight for height z-score. Adjusted for country.
